# Erdheim–Chester Disease with Isolated CNS Involvement: A Systematic Review of the Literature

**DOI:** 10.3390/neurolint14030060

**Published:** 2022-09-05

**Authors:** Anam Haque, Carlos A. Pérez, Thejasvi A. Reddy, Rajesh K. Gupta

**Affiliations:** 1 McGovern Medical School, University of Texas Health Science Center at Houston, Houston, TX 77030, USA; 2 Department of Neurology, Maxine Mesinger Multiple Sclerosis Comprehensive Care Center, Baylor College of Medicine, Houston, TX 77030, USA; 3 Division of Multiple Sclerosis and Neuroimmunology, Department of Neurology, University of Texas Health Science Center at Houston, Houston, TX 77030, USA

**Keywords:** Erdheim–Chester disease (ECD), CNS, histiocytosis

## Abstract

Erdheim–Chester disease (ECD) is a rare, sporadic, non-Langerhans cell histiocytosis that can have various presentations and higher mortality in patients presenting with neurological symptoms. We performed a systematic review to investigate and chronicle the frequency of neurological manifestations, imaging findings, treatments, and outcomes in published ECD patients presenting with neurological symptoms. A PubMed literature search was conducted for articles (published between January 1980 and June 2021) on ECD cases presenting with neurological manifestations. We analyzed the data of 40 patients, including our patient. Cranial neuropathies and ataxia were the most frequent clinical manifestations. A total of 50% of the symptomatic ECD CNS lesions were intraparenchymal and nearly 33% of patients died due to the disease itself or complications. CNS involvement may be the only manifestation of ECD and sometimes may require a repeat biopsy with IHC testing for excellent treatment outcomes.

## 1. Introduction

Erdheim–Chester disease (ECD) is a rare non-Langerhans cell histiocytosis of unclear etiology [1]. In 2016, ECD was reclassified as a hematopoietic neoplasm and it is characterized by the infiltration of tissue by foamy histiocytes with CD68 + CD1a- [1,2]. There is an uninhibited proliferation of histiocytes due to mutations in mitogen-activated protein kinase (MAP) pathways [2,3]. It has multisystem involvement, implicating long bones, the central nervous system (CNS), the eyes, the kidneys, retroperitoneum, etc. CNS involvement carries a higher rate of morbidity and mortality. Common diagnostic modalities involve imaging, including brain MRIs, tissue biopsies, and immunohistochemistry [3,4]. Surgical debulking is often required along with pharmacological treatment. Interferon alpha is the most commonly used initial treatment with the use of targeted therapies, such as mitogen-activated protein kinase (MEK) inhibitors in refractory cases [1,2,3,4].

Here, we conducted a systematic literature review of ECD cases that presented with neurological symptoms and described clinical and radiological findings, treatments, and outcomes. We also included our patient with ECD who presented with an intracranial mass requiring serial debulking.

## 2. Methods

We registered this systematic review with PROSPERO (registration number: CRD42022348565). We conducted a literature search on PubMed for studies published between 1 January 1980 and 15 July 2021 using the following keywords: “ECD CNS”, “ECD Neuro”, “Erdheim Chester Disease CNS”, and “Erdheim Chester Disease Neurology”. 

**Figure 1:**
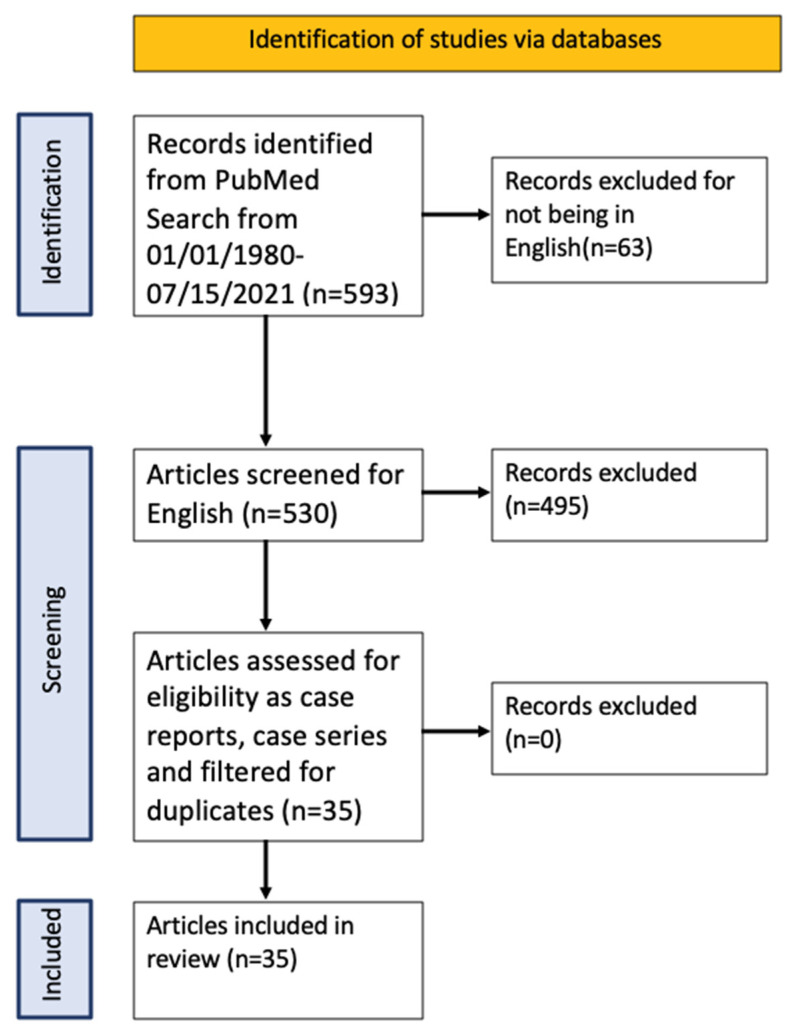
PRISMA table for the literature review.

The initial search was filtered to show articles from 1 January 1980 to 15 July 2021, yielding 593 articles (530 articles were in English). We then removed duplicates and filtered results to showcase reports and cases series of ECD patients presenting with neurological symptoms. Eligibility was assessed by reviewing abstracts and full articles where the abstracts were not available or eligibilities were not clear from the abstracts. We found 35 articles that were eligible for our systematic review (Figure 1). 

Inclusion criteria included ECD patients who initially presented with neurological symptoms. Exclusion criteria included ECD cases presenting with non-neurological manifestations, articles written in a non-English language, and articles that republished previously reported cases. We carefully evaluated each article for descriptions of neurological findings, evidence of systemic involvement, radiological findings, treatments, follow-ups, and outcomes. All of the relevant information was extracted by the lead author (A.H.). The required data were recorded, including the first author’s name, publication year, age at diagnosis, gender, treatment, and outcome. The data were verified by the last author and supervisor of this project (R.K.G.). We interpreted continuous variables as the mean with standard deviation, and categorical variables as frequencies and percentages. The level of statistical significance was set at *p* < 0.05.

## 3. Results

We retrospectively reviewed case reports and three short case series of the ECD patients who presented with neurologic symptoms and found a total of 39 cases in 35 eligible articles. After including our patient in this cohort, we analyzed the data from a total of 40 patients. In addition to demographic characteristics, we analyzed data on CNS lesion locations, outcomes, and use of various treatment modalities, including steroids, interferon alpha, cobimetinib, vemurafenib, radiation, and chemotherapy. 

Demographic characteristics and clinical data are depicted in Table 1. 

The mean age at presentation was 50.3 years with a standard deviation of 15.09. This cohort had a slight female preponderance of 52.5%. Over 52% of patients presented with cranial nerve palsy, and 50% presented with ataxia. Headaches and limb weaknesses were the subsequent most common presenting symptoms with a frequency of 28.2% each. Four patients exclusively had CNS manifestations and the remaining 36 had other system involvements. 

**Figure 2:**
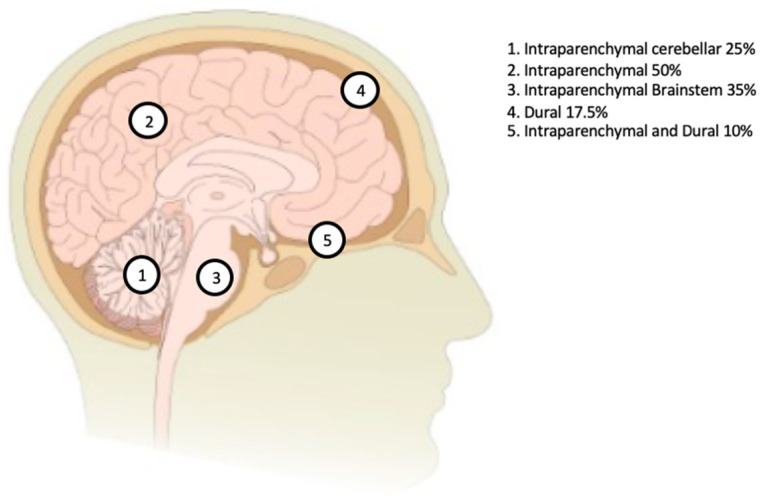
Locations of the intracranial lesions as seen on an MRI depicted on the brain diagram.

Moreover, 50% of patients had parenchymal lesions involving the cerebral hemispheres, pituitary gland, or hypothalamus; 35% had brainstem lesions, 25% had cerebellar involvement, 17.5% had dural involvement, and 10% had dural as well as parenchymal lesions found on MRIs, as seen in Figure 2. 

Over 95% of ECD patients had skeletal involvement, with approximately 50% manifesting with bone pain (Table 1). Of the 40 patients reviewed, the most commonly implemented treatment modality was steroid treatment (37.5%), 10% used interferon, 17.5% underwent surgery, and just 1 (our patient) used cobimetinib (Table 2).

In our cohort of 40 patients, 70% had imaging evidence of osseous lesions and 50% experienced bone symptoms, as depicted in Table 1. A total of 50% of patients experienced symptom and/or imaging stabilization or resolution, 32.5% experienced disease progression, and 27.5% passed away. Table 3 shows each patient’s demographic data, CNS lesion location, treatment, and outcome. 

## 4. Discussion

ECD is a form of non-Langerhans cell histiocytosis and is a clonal myeloid disease caused by activating mutations in mitogen-activated protein kinase pathways [1,2,3,4]. ECD usually presents in patients 40–70 years old (with a mean age of 53) and has a slight predilection for males [39]. Manifestations of ECD are vast, ranging from asymptomatic to mildly symptomatic bone lesions to more severe presentations involving multiple systems (Table 4).

The most common presenting symptom was bone pain, seen in as many as 95% of the patients [3]. Long bone involvement is bilateral and symmetrical and was seen in nearly all cases [5]. The vast majority of cases had imaging evidence of bony lesions on imaging, as seen in 28 of the 40 patients. Up to 50% of patients with ECD had neurologic manifestations, and these patients were shown to have higher morbidity and poorer prognoses [4]. Neuropsychiatric manifestations were seen in as many as 21% of patients and 40–70% had cardiac involvement. In a longitudinal observational study by Boyd et al., which followed 62 ECD patients, the most common presenting neurological symptoms were peripheral neuropathy (56%), cognitive difficulty (52%), cerebellar ataxia (46%), pyramidal tract symptoms (30%), and seizures (8%) (Table 4) [2,4]. 

The most common location of CNS lesions in our study was intraparenchymal, similar to locations reported in various previous cohorts in the literature, as depicted in Table 5. 

Thirty to fifty percent of ECD cases were shown to have dural changes, increased thickness of the dura mater, or nodular masses that may have been associated with parenchymal lesions [48]. Dura mater involvement can make patients susceptible to atraumatic subdural hematomas [19,48]. We found three patients in the literature with exclusive CNS involvement. One patient had a BRAF-positive parenchymal mass, which responded to vemurafenib treatment. Two other patients by Wagner et al. were BRAF-negative; one improved after debulking surgery and the other experienced disease progression despite the use of interferon and debulking [5,8]. Our patient (who was BRAF-negative) experienced progression after interferon and required serial debulking and cobimetinib therapy for disease stabilization. In Table 6, we have summarized major retrospective case series, which published data regarding common treatment modalities and prognosis of patients with ECD involving CNS. 

This review emphasizes the significance of considering ECD in differential diagnoses in patients presenting with focal CNS lesions of unclear etiology; it draws attention to the critical role of IHC testing and targeted therapy in medical management.

As this systematic review involved a retrospective literature search, it is possible that some case reports and series were missing from the literature search and were therefore not reviewed. As a result, this systematic review may not be entirely comprehensive. Additionally, due to the varied clinical presentations of ECD, it is likely that several patients with ECD and CNS manifestations were not accurately diagnosed, written up, or treated. This also contributed to cases that were not included in this systematic review. In addition, in some case studies and reviews, the patients were not followed-up with or data on their outcomes were not recorded, meaning that associations between treatments and outcomes were skewed—patients who passed away or experienced symptom resolution may have been lost to the follow-up, for example. Although these limitations have impacted this paper, the systematic review was still significant in highlighting the fact that ECD may present solely with CNS symptoms. Neurologists should be aware of this condition, which has high mortality and morbidity, especially in patients presenting with neurological symptoms.

**Figure 3:**
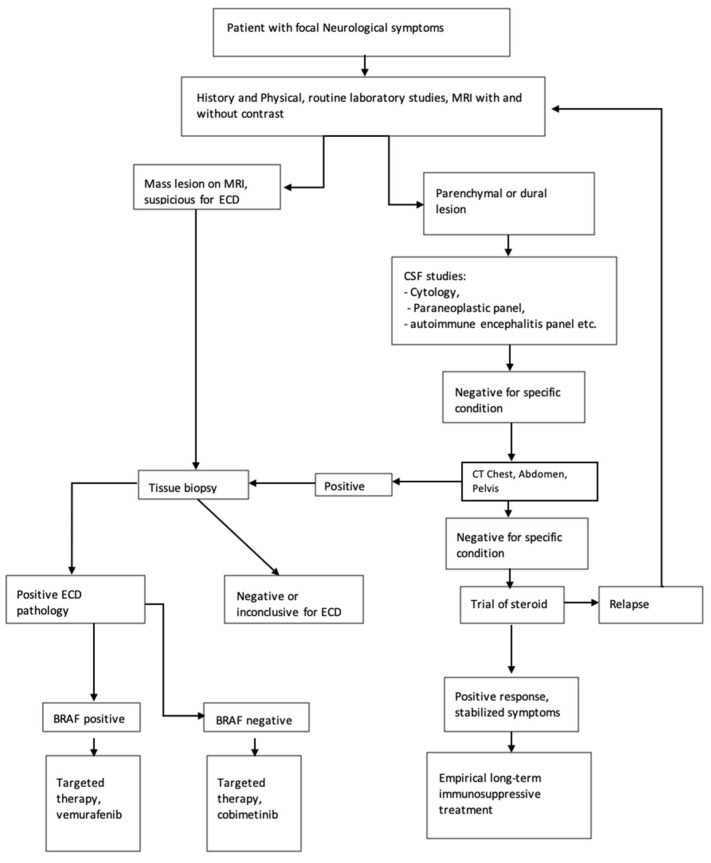
Proposed algorithm for work-up of patients presenting with focal neurological symptoms; recommended testing if ECD is suspected. Positive ECD pathology–histopathology indicative of ECD diagnosis; positive response—no further symptom progression or resolution of symptoms; stabilized symptoms—no further progression of symptoms.

In Figure 3, we propose a diagnostic and treatment algorithm for patients who present with focal neurological symptoms; ECD is considered in differential diagnoses.

## 5. Conclusions

Neurological manifestations may be the only presenting symptoms in a patient with ECD. As such, it is important to keep ECD in mind when treating a patient with a new-onset seizure, ataxia, or cognitive difficulties if an intracranial parenchymal or dural mass is found on the cranial imaging. ECD with CNS symptoms is associated with poor outcomes as well as elevated mortality and morbidity compared to ECD without neurological manifestations. Therefore, it is vital to diagnose ECD in patients who may only have neurological manifestations of disease and treat them in a timely manner. It is necessary to note that typical ECD treatments, such as interferon, may not be as effective in ECD patients presenting with CNS symptoms, or patients who have significant neurological manifestations in addition to systemic involvement. Additionally, the genotype of malignant cells also affects the response to different treatment modalities; therefore, IHC testing is necessary to guide the specific treatment [49]. 

## Figures and Tables

**Table 1:** The characteristics of ECD patients who presented with neurological symptoms.

**Characteristic**	**No, Mean (+/− SD)**	**%, (Range)**
Male	**19**	**47.5%**
Female	**21**	**52.5%**
Age and y at ECD diagnosis	**50.3 (+/− 15.09)**	**(10–75)**
Follow-up duration in months		**(1 to 144)**
**Neurological presentation**	**Frequency (Case Count)**	**Frequency (%)**
Cranial neuropathies	21	52.5%
Ataxia	20	50%
Headache	12	30%
Limb weakness	12	30%
Cognitive impairment	10	25%
Vision loss/vision symptoms	5	12.5%
Pyramidal	8	20%
Dizziness	4	10%
Asthenia	3	7.5%
Seizure	2	5%
Paresthesia/hypoesthesia	6	15%
Syncope/loss of consciousness	3	7.5%
Scanning speech	5	12.5%
Aphasia	1	2.5%
**Presence of non-neurological symptoms**	**Frequency (case count)**	**Percentage**
Bone symptoms	20	50%
Hypopituitarism	17	42.5%
Xanthelasma	8	25%

Some patients experienced symptom progression and died; some experienced symptom improvements and died; therefore, the percentages of patients who experienced specific outcomes may not add up to 100%.

**Table 2:** Treatments and outcomes of cases in this systematic review.

**Treatment**	**Number**	Percentage *
Steroid	15	37.5%
Interferon	8	20%
Surgery or debulking	7	17.5%
Vemurafenib	5	12.5%
Chemotherapy	3	7.5%
Radiation	2	5%
Cobimetinib	1	2.5%
Outcome *	**Number**	Percentage *
Improvement or stabilization of symptom	20	50%
Progression	13	32.5%
Death	11	27.5%

* Some patients received more than one form of therapy, so the percentages may not add up to 100%.

**Table 3:** Demographic data and CNS lesion location, management, and outcome of cases used for this systematic review.

Authors	Age	Gender	CNS Imaging Location	Management	Outcome
Pan et al., 2017 no. 1 [5]	47	M	C, IP, BS, B	ND	M
Pan et al., 2017 no. 2 [5]	67	F	BS	V	I
Pan et al., 2017 no. 3 [5]	46	F	IP, D, B	Ch	P
Caparros- Lefebvra et al., 1995 no. 1 [6]	74	F	IP, D, B	ND	M
Caparros- Lefebvra et al., 1995 no. 2 [6]	56	F	IP, D, B	St	I
Pineles et al., 2011 no. 1 [7]	26	F	B	St, Ch, IFN	Stab
Pineles et al., 2011 no. 2 [7]	32	F	IP, B	IFN	I
Wagner et al., 2018 no. 1 [8]	60	M	D	S	I
Wagner et al., 2018 no. 2 [8]	42	F	D	S, IFN	P
Marano et al., 2020 [9]	67	M	C, BS, B	V	I
Alvarez- Alvarez et al., 2016 [10]	74	M	IP, D	St	I
Calandra et al., 2017 [11]	42	M	IP, B	St, IFN, S	I
Bradshaw et al., 2016 [12]	52	M	BS, B	St, V	I
Jain et al., 2013 [13]	40	M	IP, B	St	I
Todisco et al., 2020 [14]	52	M	C, BS, IP, D	V	I
Viswanathan et al., 2014 [15]	50	M	IP, D	IFN	I
Mathis et al., 2016 [16]	59	F		IFN	I
Liotta et al., 2012 [17]	41	M	C, IP, B	IFN, St	I
Suzuki et al., 2016 [18]	67	M	IP, BS, B	S, St	P
Noh et al., 2020 [19]	59	F	C, IP	ND	ND
Loureiro et al., 2018 [20]	25	F	IP	ND	ND
Miron et al., 2019 [21]	55	M	C, IP, B	V	ND
Conley et al., 2010 [22]	58	F	IP	S	P
Moussouttas et al., 2021 [23]	64	M	IP	ND	P
Fargeot et al., 2017 [24]	68	F	IP, B	In	P
Rice et al., 2016 [25]	46	F	BS, B	St, PLEX	P
Black et al., 2004 [26]	51	M	IP, BS, B	ND	P
Perez et al., 2014 [27]	28	M	IP, BS, B	Ch	M
Garg et al., 2021 [28]	44	F	C, IP, BS, B	St	M
Sagnier et al., 2016 [29]	64	M	B	infliximab	M
Rodrigues et al., 2021 [30]	42	F	IP	St, IFN	Stab
Johnson et al., 2004 [31]	34	M	IP D, B	R	Stab
Jeon et al., 2021 [32]	75	F	BS, B	S	Stab
Kumandas et al., 2007 [33]	10	M	IP, D, B	St	ND
Fukazawa et al., 1995 [34]	59	F	C, B	ND	P
Bohlega et al., 1997 [35]	37	F	IP, BS, B	R	Stab
Evidente et al., 1998 [36]	69	M	C, BS, B	St	I
Wright et al., 1999 [2]	42	F	C, BS, B	St	I
Pego- Reigosa et al., 2000 [37]	50	F	D, B	St, S, R	ND
Haque et al., 2022 [38]	38	F	IP, D	IFN, S, C	Stab

IP—intraparenchymal; BS—brainstem; D—dural; C—cerebellar; B—bone; S—surgery; St—steroids; IFN—interferon, pegylated interferon; Ch—chemotherapy; R—radiation; V—vemurafenib; C—cobimetinib; PLEX—plasma exchange; I—improvement; P—progression; M—mortality; Stab—stabilized; ND—not documented.

**Table 4:** Frequency of CNS, bone, and other system involvement in ECD as reported in the literature.

Publication.	No. Patients/Article Type	CNS Symptoms (%)	Bone Symptoms (%)	Other Symptoms (%)
Cives et al., 2015 [1]	448, RCS	55.6% (23.2% visual, 21.8% ataxia, 9.8% dysarthria, 7.1% para or hemiparesis)	74.1%	36.2% retroperitonea l10.7% cardiac 26.8% skin
Pegoraro et al., 2020 [40]	360	39%	89%	65–75% with retroperitoneal 40–45% cardiac 25% diabetes insipidus 25–50% lung
Cavalli et al., 2013 [41]	259	51%	* 50%	30% retroperitoneal 25% diabetes insipidus 22% cardiac
Haroche et al., 2004 [42]	72	35%	* 100%	100% cardiovascular 35% diabetes insipidus 44% exophthalmos
Boyd et al., 2020 [4]	62	94% (52% cognitive, 61% cranial neuropathy, 56% peripheral neuropathy, 46% cerebellar ataxia)		22% proptosis
Estrada- Veras et al., 2017 [43]	60	92% (56% peripheral neuropathy, 48% cognitive, 40% cerebellar ataxia, 23% headache, 15% diplopia, 14% dysarthria)	95%, (50% with bone pain)	62% coated aorta 65% retroperitoneal 47% diabetes insipidus 30% restrictive lung pattern of breathing 25% xanthelasma
Arnaud et al., 2011 [44]	53, RCS	51%	96%	68% retroperitoneal 64% with cardiac involvement 28% with cutaneous involvement
Drier et al., 2010 [45]	33, RCS	45% (17% ataxia, 9% seizures, 9% panhypopituitarism)		24% diabetes insipidus 21% exophthalmos
Starkebaum, Hendrie, 2020 [3]	Research article	50%	95% (symptomatic in 50%)	47% Diabetes insipidus

RCS—retrospective case series. * inclusion criteria included bone involvement.

**Table 5:** Common sites of CNS lesions in ECD as reported in the literature.

Publication	No. of Patients, Report Type	Brain MRI Findings
Bhatia et al., 2020 [46]	30 patients; retrospective review involving patients who presented with neurological symptoms; single institute study	60% with parenchymal lesions 33% with dural involvement
Lachenal et al., 2006 [47]	6-patient case series with CNS involvement; a systematic review of 66 patients with CNS involvement	44% with parenchymal lesions 37% with dural involvement 19% with parenchymal and dural lesions
Arnaud et al., 2011 [44]	53 patients; prospective cohort	43% with diencephalic involvement 17% with dural involvement
Drier et al., 2010 [45]	33 patients; retrospective review	47% with hypothalamic–pituitary axis involvement 23% with dural involvement
Boyd et al., 2020 [4]	62 patients with ECD were prospectively enrolled in a natural history study	50% with brain parenchymal lesions 6% meningeal involvement
Estrada- Veras et al., 2017 [43]	60 patients; prospective cohort	36% with parenchymal lesions 7% with meningeal involvement

**Table 6:** Treatment and prognosis of ECD patients involving CNS, as reported in the following retrospective studies in the literature.

Publication	Number of Cases, Report Type	Treatment	Prognosis
Lachenal et al., 2006 [47]	66, RCS	73% steroids 43% chemotherapy or immunosuppressants 29% radiotherapy 18% underwent surgical treatment	10% stabilized 42% progressed 48% died
Estrada- Veras et al., 2017 [43]	60, RCS	33% IV methylprednisolone 27% IFN alpha 12% anakinra	IFN alpha: 78% stabilized 17% progressed Anakinra: 57% stabilized 43% progressed Methylprednisolone data not available
Arnaud et al., 2011 [44]	53, RCS	57% steroids 87% interferon 42% chemotherapy or immunomodulatory therapy	96% 1-year survival rate 68% 5-year survival rate
Bhatia et al., 2020 [46]	30, RCS	10% radiotherapy 24% conventional therapy—steroids, immunomodulatory therapy, IFN alpha, and chemotherapy 64% conventional therapy followed by targeted therapy, such as a BRAF inhibitor, MEK inhibitor, or combined BRAF/MEK inhibitors	With conventional therapy: 67% experienced progression 19% stabilized 14% experienced complete resolution With targeted therapy, 85% experienced partial or complete resolution of symptoms

RCS—retrospective case series.
